# Using critical slowing down indicators to understand economic growth rate variability and secular stagnation

**DOI:** 10.1038/s41598-020-66996-6

**Published:** 2020-06-26

**Authors:** Craig D. Rye, Tim Jackson

**Affiliations:** 1 Climate Science Awareness and Solutions, Columbia University (CSAS), New York, USA; 2 Centre for the Understanding of Sustainable Prosperity (CUSP), University of Surrey, Guildford, UK

**Keywords:** Applied physics, Environmental social sciences

## Abstract

This paper utilizes Critical Slowing Down (CSD; instability) indicators developed by statistical physics to analyse economic growth rate variability and secular stagnation in historical GDP data. Understanding these phenomena is vital, particularly in advanced economies faced with declining growth rates. Two novel indicators - the autocorrelation (AR1) and the variance – are found particularly useful in providing insight into inter-decadal GDP variability over this period. These indicators are first applied to the Maddison-Project historical dataset, which includes almost a century of data for some 80 countries and almost two centuries of data for 9 countries. They are additionally applied to ~50 years of recent annual data for around 130 countries from the World Bank dataset as well as ~60 years of recent quarterly data for around 20 countries from the OECD dataset. Analysis reveals inter-decadal variability in growth cycles (the recession cycle), highlighting periods of large slow growth cycles and periods of small fast growth cycles. The most commonly occurring pattern is characterised by an increase in CSD from the 1900s to 1940s, a decline in CSD between the 1930s and the 1970s, then a further increase in CSD from the 1960s to 2010. This pattern is significant in ~70% of the advanced economies. CSD indicators may then provide invaluable insights into specific aspects of inter-decadal GDP variability, such as on the nature of the business cycle, secular stagnation and the implicit “restoring forces” of the economy.

## Introduction

The economic growth rate (as measured by the Gross Domestic Product or GDP) has declined across many advanced economies over recent decades^1–5^. Over the same period, GDP time series express a diverse range of annual to multi-decadal variability, commonly discussed in terms of recession cycles. Our understanding of how this variability in GDP has changed and its importance for long-run trends is poor. Novel analysis borrowed from the physical sciences may provide useful new insight. Our work is particularly motivated by an interest in the biophysical inputs to the economy and their role in the slowing down of economic growth^6–11^.

The recent decline in economic growth is most apparent in the G7 block and is particularly prominent amongst USA, UK, Japan, Italy, France, and Germany, where the decadal averaged growth rates have dropped from around 4% in the 1970’s to around 2% in recent years^5^. This decadal growth reduction is notably referred to as ‘secular stagnation’ by Summers (2013)^2^ and in the literature^12^. It is perhaps most prominent in the case of Japan whose growth rate has moved from around 10% in 1970 to around 2% in 1990, remaining at around 2% thereafter. In addition in the most recent decade, even large emerging economies such as China and India have also experienced a steady decline in growth from around 10% in 2007 to around 7% in recent years.

A wide range of ‘head-winds’ or inter-decadal drivers of secular stagnation are discussed by the literature, including public debt overhang, reduced aggregate demand, reduced innovation (supply), policy uncertainty, changes in demography, decline in education attainment growth, increased inequality, decline in labour productivity growth, decline in the quality of primary resources and changes in the structure and organisation of the financial sector^2,3,5,13–16^. The suggestion of Reinhart and Rogoff (2009)^13^ that public debt overhang is the main driver of stagnation appears to have played an important role in the initial government policy responses following the financial crisis of 2008. However, there is no clear consensus on the driving mechanisms of stagnation^16^.

Recent literature suggests that cycles in the GDP growth rate (i.e. oscillations in de-trended GDP, also referred to as recession cycles) have a characteristic timescale of around 10 years^17^; however, historical literature discusses a wide range of recession cycle timescales^18–22^. Here, four timescales are highlighted to illustrate this diversity. Kitchin (1923)^18^ describes a short 3–5 year timescale and argues that it is related to the renewal of business inventories. Juglar (1860)^19^ describes a medium 7–11 year timescale and relates it to business capital investments. Kuznets (1930)^19^ discusses a 15–25 year scale and links it to infrastructure investment. Finally, Kondratieff (1979)^22^ describes a 45–60 year scale and relates it to technological change. Despite their recognition, little consensus exists on the driving mechanisms for these scales^23–25^. Further, there is little analysis on the interaction or inter-decadal variability of these scales.

Kuznets and Kondratieff scale variability are of particular interest because of their likely importance for inter-decadal macro dynamics. Kondratieff-scale variations are commonly referred to as k-waves (or long-waves) and have been associated with a number of forcing mechanisms, from political upheaval to resource availability^25^. Interestingly, Kuznets scale variability is additionally associated with inter-decadal (secular) trends in inequality, and with the Kuznets-curve, which implies a relation between GDP per capita and inequality^26^.

**Figure 1:**
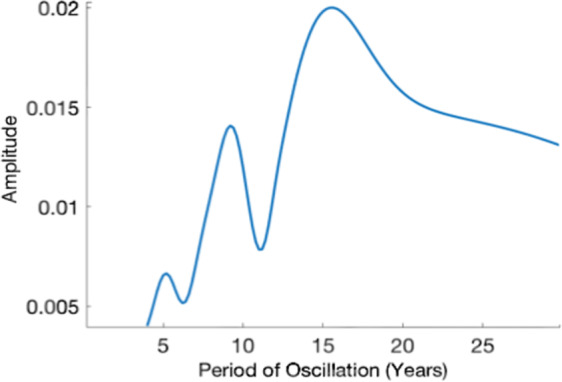
Fourier decomposition of the UK GDP per capita data 1960-present. Data are taken from the Maddison Project databas^44^.

Preliminary analysis conducted by the author’s additionally suggests that recession cycles occur on a wide range of timescales. This variability is well illustrated by a Fourier decomposition of a de-trended GDP series (Fig. 1), as described by Eq. 1.

1 $$\widehat{GDP}(f)=\,\int \mathop{GDP}\limits^{\cdot }(t){e}^{-2{\rm{\pi }}{\rm{ift}}}dt$$

where $\mathop{{\rm{GDP}}}\limits^{\cdot }$ denotes the de-trended series, t is time, f is frequency. Where the given time series is decomposed into its constituent frequencies and expressed in terms of their relative importance in the series. Figure 1 shows the Fourier decomposition of UK GDP variability between 1960 and 2016. The transform highlights the dominance of variability on 5, 9 and 17-year timescales.

As suggested by Fig. 1, analysis techniques borrowed from the physical sciences may provide novel insight into GDP variability and secular stagnation. Although multiple authors have analysed the relationship between the GDP growth rate and de-trended GDP variability, they have tended to focus on short timescale phenomenon (around 10 years). For example, Ramey and Ramey (1994)^27^ and Hnatkovska and Loayza (2005)^28^, both provide evidence of a negative correlation between economic growth and its volatility. Reinhart and Reinhart (2015)^29^ found a negative correlation between the magnitude of crises leading to a recession and the growth rate following a recession. Further analysis of long-term GDP variability is particularly timely not just in light of the continuing ‘headwinds’ widely associated with the financial crisis, but also because of growing concerns around economic instability associated with long-term policy issues such as climate change, resource depletion and biodiversity loss^6,10^. Following Reinhart and Reinhart (2015)^29^, a high-quality indicator of GDP instability could provide invaluable insights in to multi-decadal macro trends such as secular stagnation.

Despite instability indicators being an active topic of research, however, there is little consensus on their utility. For example, macro-scale indicators of instability^30^ have been constructed from aggregates of micro level indicators of financial viability (e.g. Altman’s Z-score^31,32^). But these indicators are imperfect, as they do not account for firm-firm and firm-institution dynamics^33^.

Novel data analyses techniques, borrowed from the complex systems sciences, have been used to explore economic instability. For example, Ramirez and Rodriguez (2011)^34^ have analysed the Dow Jones Index (DJI) in terms of the variability of its ‘entropy’, where they observe a 22-year cycle in the DJI data. Quax *et al.* (2013)^35^ have analysed variability in the ‘information dissipation length’ of interest rate swaps (IRS), finding early warning signals in the period leading to the Lehman Brothers collapse. Tan and Cheong (2014)^36^ have analysed the dynamics of instability in the U.S. housing market. Furthermore, Diks Hommes and Wang (2015)^37^ use the time-variability of variance and autocorrelation (auto-regression) to quantitatively the instability of financial indices. These novel techniques provide useful new insights. But they tend to deal with relatively short time-scales and mainly with financial indicators. Our aim in this paper is to employ stability indicators from the natural sciences to analysis, in particular, multi-decadal variability in the GDP.

### Critical slowing down

Our analysis is based on the theory of Critical Slowing Down (CSD) in physical systems and on the use of the instability indicators (in particular, first order autoregressivity (AR1) and variance) employed by Diks Hommes and Wang (2015)^37^ and others^38^. These are derived in their turn from the theoretical work of Wiessenfeld (1985)^39^ and Wissel (1984)^40^. CSD theory is sufficiently general that it can be applied to any time-periodic system comprised of deterministic and stochastic components. CSD theory has been applied to a wide range of datasets in various fields of research, including Climate Physics^41^ and Finance^37^; however, CSD analysis has not been previously used to examine national macro datasets such as GDP. CSD analysis provides a measure of the change in the characteristics of variability as highlighted by spectral analysis and suggested by the literature^18–20^ (Fig. 1). Preliminary work (by the authors) suggests that CSD behaviour may be present in GDP time-series (Fig. 1).

**Figure 2:**
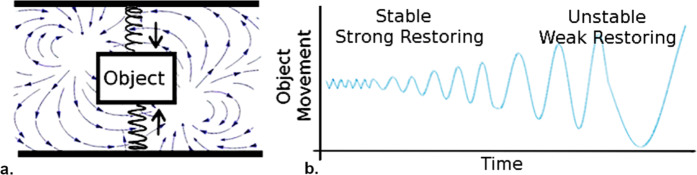
(**a**) Schematic of the spring-object-spring system. The restoring forces of the spring: black arrows. The stochastic forcing of the the winds: blue arrows. (**b**) Schematic of the CSD behaviour of the spring-object-spring system, as the strength of relative restoring forces is diminished.

CSD behaviour as outlined by Wiessenfeld (1985)^39^ and Wisel (1984)^40^ is best described with an abstract example of an oscillating system. Consider an object that is suspended between two springs, in a windy environment (the spring-object-spring system; Fig. 2a). The springs provide a deterministic restoring force (black arrows), and the wind provides a series of stochastic perturbations. As the wind pushes the object away from its equilibrium position the springs pull the object back, leading the system to oscillate about its equilibrium. If the springs are strong (strong restoring), the oscillations are fast and small. If the springs are weak (weak restoring), the oscillations are slow and large. If the relative strength of the springs becomes weaker over time, then the oscillations of the system transition from small and fast to large and slow (Fig. 2b). This transition is characteristic of CSD behaviour. An additional example is given in Supplementary Material.

A premise of our own study is that CSD theory may also be applicable to economic systems. In the case of a national economy, for example, restoring forces might include the relationship between profits and wages, the relationship between investment and discretionary spending, or the price mechanism for commodities. In the next section, we describe some of the specific quantitative methods used in the literature to identify CSD.

### Autocorrelation analysis for CSD

Before analysis, time series must be de-trended to isolate variability about the equilibrium growth trend. In our study, data are de-trended using a Hodrick-Prescott filter (HPF) with an averaging windows ranging from 10–30 years. The choice of de-trending is discussed further in the methods section.

A widely used indicator of CSD is the AR1 autocorrelation^2,42^. This is in essence, the correlation of a time series with itself lagged by one time-step. AR1 is estimated in a moving window as described by Eq. 2. A typical window length for estimating AR1 is 15 years. The significance of the AR1 indicator is tested using a range of window sizes between 5 to 40 years (see Methods).

2 $$AR1{\left(t+\frac{v}{2}\right)}^{n}=\frac{1}{v}\mathop{\sum }\limits_{\hat{t}=t+1}^{\hat{t}=t+v}\frac{(GD{P}_{(\hat{t})}^{n}-{{\rm{\eta }}}_{(\hat{t})}^{n})(GD{P}_{(\hat{t}-1)}^{n}-{{\rm{\eta }}}_{(\hat{t}-1)}^{n})}{{(GD{P}_{(\hat{t})}^{n}-{{\rm{\eta }}}_{(\hat{t})}^{n})}^{2}}$$

ν is the chosen window size, ${\rm{\eta }}$ is the value of the model used to de-trend the data or the local mean ${{\rm{GDP}}}^{n}$ ($GD{P}_{(\hat{t}-1)}^{n}-{{\rm{\eta }}}_{(\hat{t}-1)}^{n}$ is equal to $\dot{GDP}$). n denotes the given nation, $\hat{t}$ is the time variable inside the moving window.

### Variance analysis for CSD

The change in the variance is also widely used as an indicator for CSD^39–41^. Low variance suggests that the system oscillations are small, high variance suggests that oscillations are large. The variance is estimated using the same de-trending method and moving window as the AR1 indicator, described in the previous section as shown in Eq. 3.

3 $$Var{\left(t+\frac{v}{2}\right)}^{n}=\frac{1}{v}\mathop{\sum }\limits_{\hat{t}=t}^{\hat{t}=t+v}{(GD{P}_{(\hat{t})}^{n}-{{\rm{\eta }}}_{(\hat{t})}^{n})}^{2}$$

Any trend in variance is likely to be biased by the growth of an economy. i.e the magnitude of the oscillations of an economy is likely to be equated to its size; where a larger economy is likely to have large oscillations. The simplest correction for this is to express GDP variance as a percentage of the GDP (discussed below). Further work is required to optimise this correction. The variance indicator is tested for significance using the same method as described for AR1 (see Methods). A positive (increasing) trend in the AR1 and variance indicators suggests that the oscillations of the system are changing from small and fast to large and slow. In other words, in a period of CSD, the typical time-period and magnitude of variability of the system is getting larger and the strength of the system’s restoring forces are diminishing. CSD theory suggests that a tipping point is most likely to occur as the autocorrelation function approaches 1^42,43^. At this point, the oscillations of the system are exceptionally large and slow and the system may change rapidly into a new state. However, the magnitude of AR1 is strongly affected by the method of de-trending and choice of calculation window. It is therefore suggested that trends in AR1 and variance are more insightful than the absolute magnitude of these variables^41^.

## Results

### AR1 and variance applied to historical data

The AR1 and variance indicators are used to explore the Madison Project dataset of yearly per capita GDP. Data are available for 9 countries between 1820 and 2016, for 20 countries, between 1900 and 2016, and for around 80 countries between 1930 and 2016 (The Maddison-Project 2013^44^).

**Figure 3:**
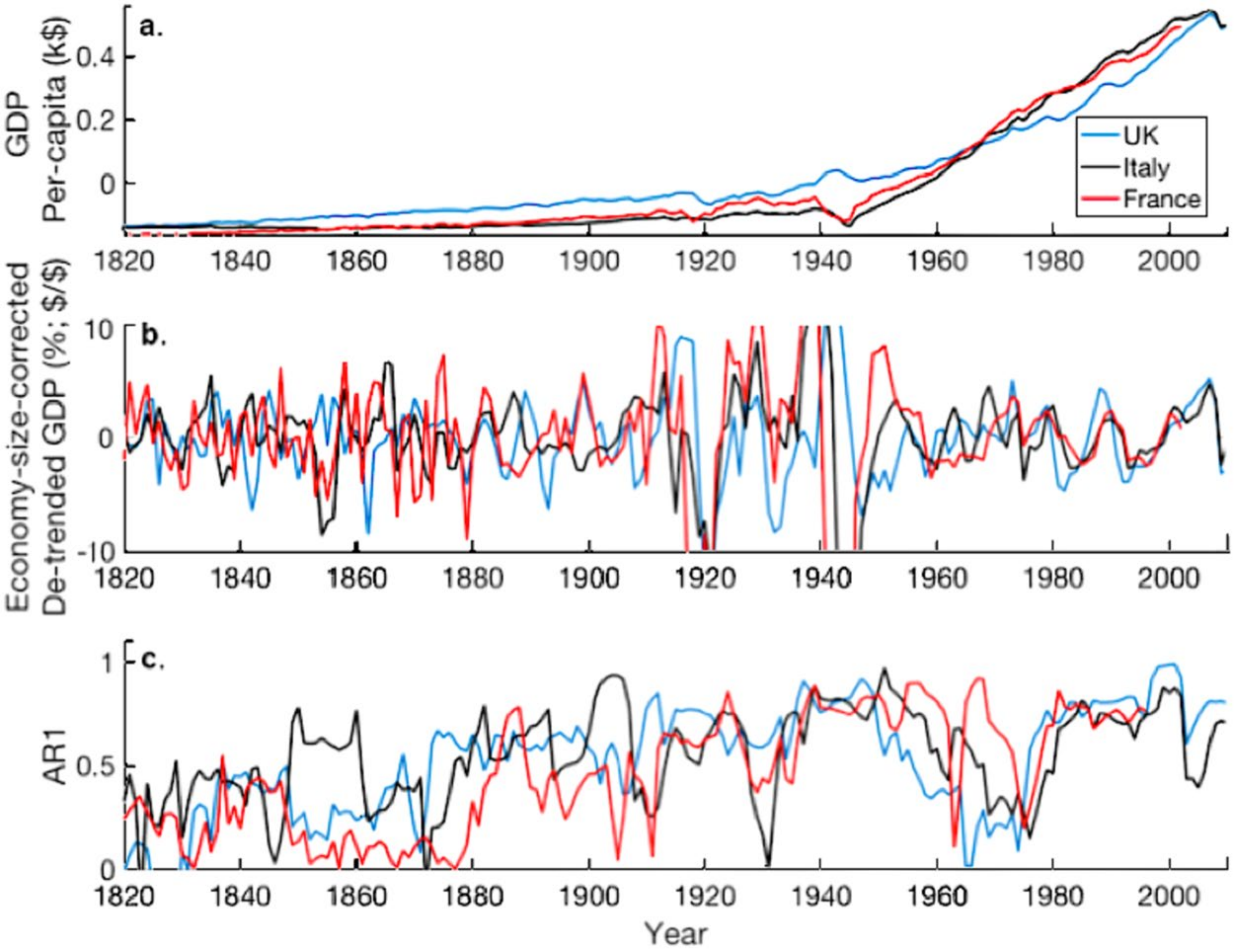
Historical CSD GDP variability for three western European economies UK, France and Italy. (**a**) UK, France and Italy GDP per capita. (**b**) UK, France and Italy GDP data de-trended using HPF filter with 30 year averaging and AR1 moving window of 15 years, the variability is economy size corrected by expressing it as a % of the overall GDP, units are arbitrary. (**c**) AR1 for historical GDP of UK, France and Italy. Units are arbitrary. Data are taken from the Maddison Project database^44^.

The extended dataset, between 1820 and 2016, is available for USA, UK, Denmark, France, Italy, Holland, Sweden, Australia and Indonesia (Fig. 3). These data highlight periods of high and low AR1 and variance, with typical time scales of 20–60 years. No clear internationally dominant pattern is apparent pre-1900. However, the early data for western European economies show a significant positive trend in AR1 between 1820 and 1950.

**Figure 4:**
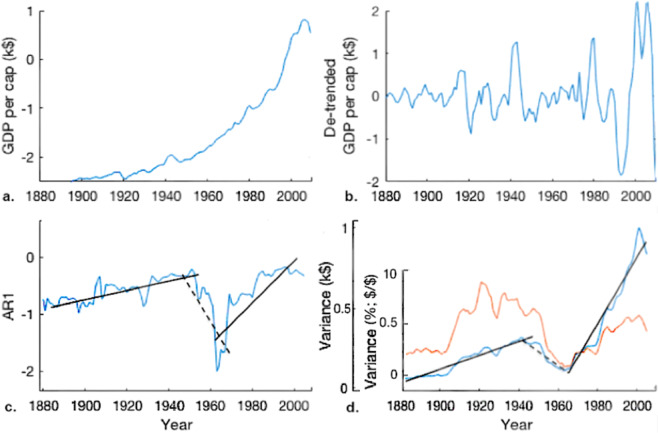
(**a**) UK GDP per capita 1880–2010, taken from the Maddison Project database^44^. (**b**) 30-year HPF de-trended UK GDP per capita; (**c**) The dominant pattern in AR1 autocorrelation for 1880–2010, UK example; (**d**) Blue line: The dominant pattern in variance (not growth corrected) for 1880–2010, UK example. Red line: The dominant pattern in economy size corrected variance.

**Figure 5:**
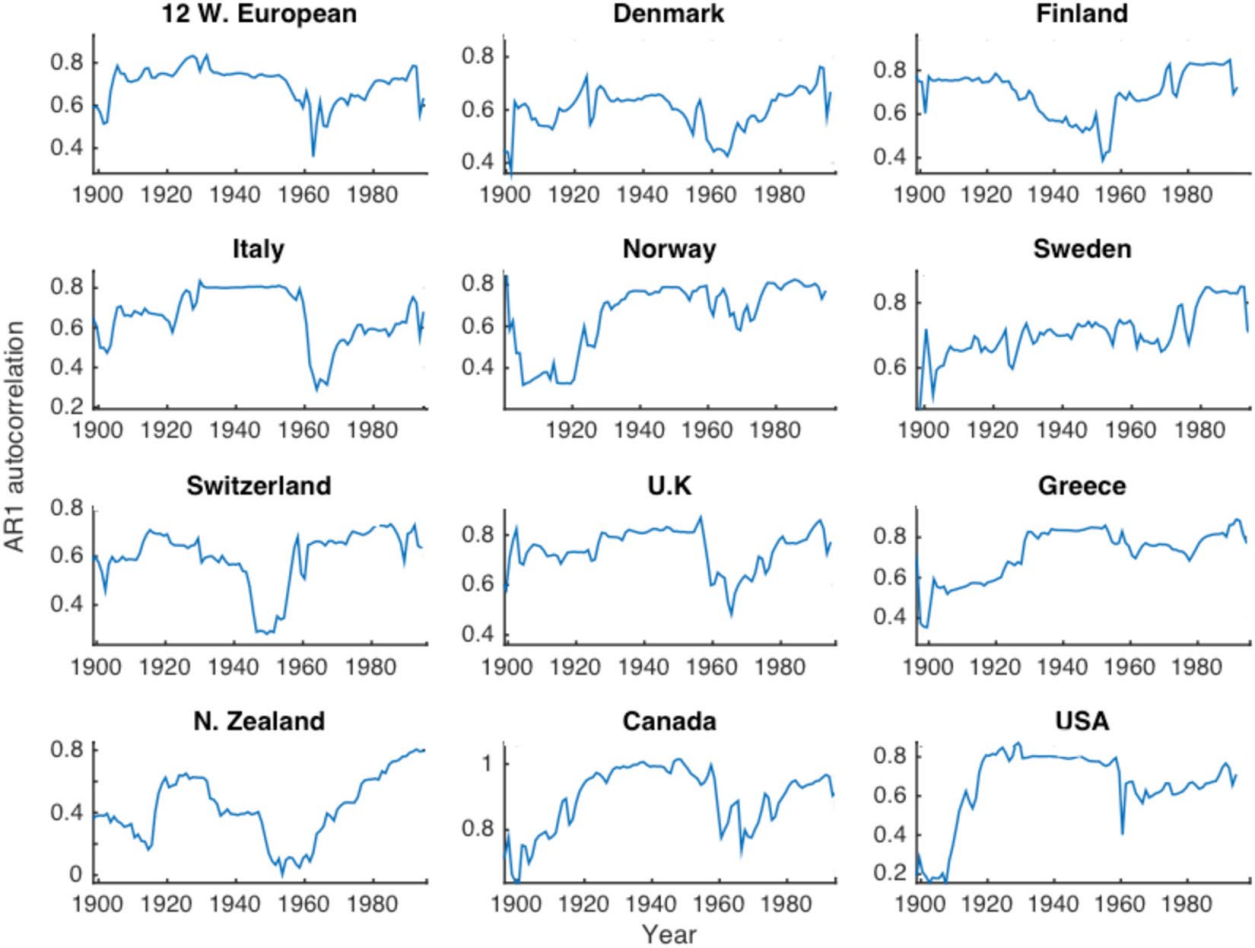
AR1 autocorrelation derived from de-trended GDP per capita data between 1900–2010, taken from the Maddison Project database (The Maddison Project 2013).

Following 1900, a dominant pattern in AR1 and variance between 1900 and 2016 is evident, shown in Fig. 4 for the UK and Fig. 5 for multiple nations. This pattern is best described as an increasing trend (+CSD) leading up to a period of instability between 1915–1950, a decline (-CSD) from 1940 leading to a period of high growth around 1960–1970. This is then followed by a second increase (+CSD) from 1950 to present day. The period of high instability between 1915 and 1950 notably encompasses the World Wars and the Great Depression.

This dominant pattern is observed in around 70% of high GDP countries and 5% of low GDP countries. It can be characterised by three linear fits, as shown in Fig. 4c,d. These fits are significant for around half of high GDP time series. Notable countries that fit this pattern significantly include, the U.K, USA, Canada, France, Germany, Denmark, Italy, Holland, Sweden (Fig. 5). At least two components of this fit are significant for around 75% of high GDP countries.

The economy-size-corrected (ESC) variance indicator suggests that the recent increase in ESC variance is around half of that found in the period of conflict in 1920–1940’s. The uncorrected variance pattern suggests the recent increase in variance is larger (Fig. 5).

The AR1 and variance indicators are additionally applied to recent yearly GDP per capita data, between 1960 and 2018, for around 130 countries, provided by the World Bank^45^. A significant trend (p < 0.05) in AR1 is found in approximately 60% of countries. 40% show an increasing trend, 20% show a decreasing trend (See Supplementary Material). The increasing AR1 group contains many large western economies such as, the United States, Canada, Australia, the United Kingdom, and Italy. It also contains large eastern economies, such as China, Indonesia and India (a list is given in Supplementary Material).

Finally, the AR1 and variance indicators are applied to the recent quarterly per capita GDP data, between 1960 and 2018, for 20 countries (mainly high GDP nations), provided by the OECD^46^. A significant trend in AR1 was found in approximately 90% of countries. 70% showed an increasing trend, 20% showed a decreasing trend (See Supplementary Material).

## Discussion

The findings of this study suggest that CSD indicators may be useful for exploring inter-decadal macro dynamics. For example, the behaviour of the spring-object-spring system described in the introductory section is qualitatively similar to the behaviour of many non-equilibrium growth models. These include the Goodwin cycle^24^, Minsky’s financial instability hypothesis^47^ and the Minksy-models researched by Keen (2013)^48^ and others. CSD indicators have also been shown to be useful for several other fields of research^38,42,49,50^.

It is important to clarify what exactly is meant by ‘instability’ in the context of analysing CSD in GDP time series. Specifically, it does not refer to variability in the GDP per se. Rather, a state of instability refers to a state of relatively slow, large, recession cycles. It can be thought of as a state of weak restoration to the equilibrium growth path or a period where an economy takes a relatively long time to return to its equilibrium growth trend, following a perturbation. CSD theory suggests that a movement from a stable to an unstable state implies the onset of a ‘critical transition’ or tipping point^41^. However, a clear tipping point, following a period of CSD, is not apparent in any of the GDP time series examined in this study. Following Kéfi *et al.* (2013)^51^, instability indicators may also be viewed as indicators for changes in the behaviour, interactions, inputs, or structure, of an economy.

Here, the term ‘instability’ spuriously implies an undesirable system state, i.e. counter to a notion of prosperity. However, it is unclear whether periods of ‘stability’ or ‘instability’ (in this context) are optimal for the wellbeing or prosperity of a society. For example, there are multiple inter-decadal trends and significant events that have occurred in the same time period as the observed trends in AR1^3^. For the recent decades, these include the decline in the quality of energy resources^52^ associated for instance with the move towards unconventional oil and gas reserves^53^ as well as political changes, demographic shifts^53^, trends in labour productivity^8,54^ and clusters of technological innovation^19^. For historical data, these include the international conflicts of 1914–1945 (WW1&2) and the great depression of 1929.

There are therefore many opportunities for long-term trends to reinforce each other and thus to facilitate unintuitive outcomes, such as prolonged recessions that reduce inequality^26^ or international conflicts that shift macroeconomic aggregates such as employment, investment and the value of the capital stock. Such changes may also provoke spurious correlations. Furthermore, it is noted that in a high GDP nation, GDP is a poor indicator of personal or societal wellbeing^9,55^. It is evident therefore that great care must be taken in the interpretation of the results of analyzing the GDP using CSD instability indicators. It should be stated that the de-trending process before CSD analysis is non-trivial and the choice of timescale used to de-trend time series can greatly alter results. Previous empirical work such as spectral analysis (e.g. see methods), suggests that emphasising a 7–11 year timescale is unjustified. The application of a conventional 7–11 year timescale, for example, appears to remove information (see methods section). Our analysis uses a 30-year time averaging time-scale; however, qualitatively similar results can be found using a 60 year averaging or by linear fit.

The most robust result of our analysis is the observation of a dominant pattern in AR1 and variance in historical data. This pattern implies an increase in instability between 1850 and 1950, a decline in instability, between 1940 and 1970, then a second increase from 1960 to 2000. This pattern is most apparent for the large GDP nations, particularly the United States, the United Kingdom, Canada, New Zealand and Ireland. The consistency of this pattern in the AR1 and variance indicators (Figs. 3–5 and Supplementary Material) is highly supportive of its validity and indicative of the validity of the general method.

**Figure 6:**
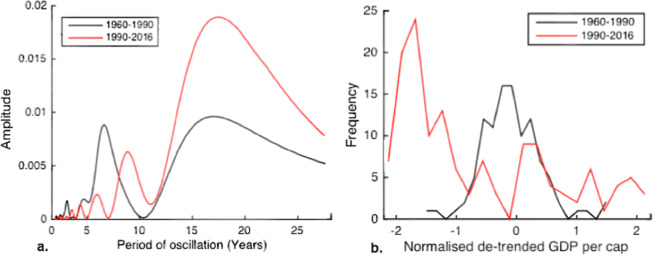
(**a**) Fourier decomposition of the UK GDP per capita data showing the spectral reddening associated with CSD. Black line: first half of a time series. Red line: second half of a time series. (**b**) Histograms of normalised de-trended UK GDP per capita 1955–2015, highlighting a change in skewness of GDP anomalies. Red line: 1990–2015. Black line: 1960–1990. An equivalent plot for spectral reddening is shown in Fig. 1. Data are taken from the Maddison Project^44^.

In addition, three supplementary CSD indicators, spectral reddening, skewness and inter-correlation, are found to support the dominant pattern observed in AR1 and variance. Spectral reddening analysis (Fig. 6a) shows the change in frequency of a system’s oscillations^40^ and Skewness analysis (Fig. 6b) shows the change in the symmetry of oscillation cycles^41–43^. CSD theory suggests that a system’s oscillations become ‘redder’ and more asymmetric as it tends towards instability^42,43^. Both analyses show a comparable increase in the dominant time-scale and skewness of de-trended GDP variability from 1900 to 1930 and from 1960 to present. Spectral reddening and skewness analysis treat each oscillation as a single observation and therefore greatly reducing the resolution of data. They are useful in characterising the kind of variability occurring in decadal blocks but do not provide sufficiently high resolution to warrant primary results.

**Figure 7:**
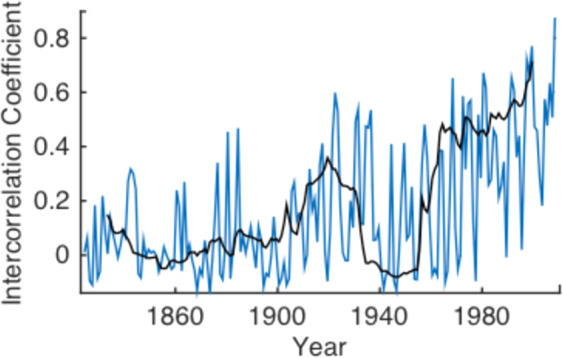
The inter-correlation coefficient between USA, UK, Denmark, France, Italy, Holland, Sweden, Australia and Indonesia, taken from the Maddison Project databas^44^. The black line is calculated with a moving window of 30 years. The blue line is calculated with a window of 5 years.

Inter-correlation analysis provides a measure of the CSD expressed by the global economy. This is also referred to as the inter- or intra-correlation coefficient (ICC). This indicator is estimated for the Maddison project historical per capita GDP data, for the 9 countries with the longest available time series (USA, UK, Denmark, France, Italy, Holland, Sweden, Australia and Indonesia; 1820–2010). The result is shown in Fig. 7. A similar pattern occurs to that observed for the AR1 and variance indicators (Figs. 4 and 5).

It is clear that historical GDP oscillations or recession cycles occur on a range of time scales from 5 to 30 year. Further, there are historical periods where GDP variability is dominated by typically slow-large cycles indicative of Kuznets and Kondratieff -scale variability and periods dominated by typically fast-small cycles supportive of Jugler-scale variability. Complex systems theory suggests that the move of an economy between different dominant scales of variability is indicative of changes in the structure of an economy or in the economic climate^40–42^. Further work is required explore the structural changes that may be associated with variability in the characteristics of the recession cycle.

In general, the results of our analysis relate to multiple discussions on long-term macroeconomic dynamics. Notable examples include: the arguments from k-wave theory that highlight a repeating long-term (20–60 year) cycle in GDP growth rate^25^; the work of Reinhart and Reinhart (2015)^29^ who highlight an inverse relationship between economic crises (GDP volatility) and growth; a study by Harras and Sornette (2008)^56^, proposing that market instability develops endogenously by a self-organizing market process, during periods of low growth; and the work of Minsky (1992)^47^ and Keen (2010)^48^, who argue that instability develops endogenously in financial systems. Finally, our results may relate to the work of Brucker and Gruner (2011)^57^, who find a link between declining growth rates and political instability. Extensive work is required to understand the results of CSD analysis in the context of the literature.

An important concern of our analysis is the relationship between CSD and the growth rate of an economy. Douthwaite (1992)^55^ and others have argued that growth is necessary for the stability of a capitalistic economy, particularly^58^ where this entails a debt-based money system. Jackson and Victor (2015)^59^ showed that a quasi-stationary economy with debt-based money and a long-term zero trend in the growth rate is formally possible. But several authors have highlighted potential instabilities that arise when a capitalistic economy slows down^8,56^.

Our analysis suggests that there may be an inverse relationship between the AR1 indicator and the GDP growth rate. However, this relationship is variable and difficult to quantify. This relationship is most apparent in the aggregate per capita GDP datasets, such as the OECD aggregate, the G7 aggregate and the Western Europe aggregate. These aggregate time series express a strong negative (inverse) correlation between AR1 and the GDP growth rate, between 1900 and 2016. This relationship is more prominent in the recent (1960–2016) national per capita GDP datasets, for around 40% of recent countries and specifically 60% of high GDP national datasets.

At the time of submitting this manuscript, the global economy is facing one of the largest downturns since the Great Depression in the 1930s, in response to the COVID-19 pandemic. This represents an exceptional exogenous shock that will likely drive a deep recession cycle of exactly the kind analysed in this manuscript. Our analysis indicates that, preceding the crisis many advanced economies were already in a state of high CSD characterised by large slow growth cycles (recession cycles). CSD theory then suggests that a post-coranavirus recovery could be characterised by weak restoring forces and a slow return to equilibrium. On the one hand, this presents a clear threat to the structural stability of the global economy. On the other, it may offer opportunities for economic restructuring that would not have been possible in a climate of stronger restoring forces characterised by lower CSD.

Ultimately this work is inspired by an interest in long-term macro dynamics and global challenges, such as climate change. An optimal mitigation strategy for environmental challenges may lead to a decline in economic output, even if this is associated with higher wellbeing. Many authors have highlighted that a long run decline in GDP does not necessitate a decline in living standards, particularly for the high GDP per capita nations^8,60–62^. It is possible that aggressive climate change mitigation strategies aimed at achieving international climate targets will create a downward pressure on the GDP growth rates (Stern 2006; Jackson 2017). Regardless of this, climate change and biodiversity loss will inevitably lead to long-run declines or even a collapse in economic output if left unaddressed^6,7,63^. In summary, our understanding of the national and international dynamics of a declining political-economy is poor. It is clear that intensive research is required to inform long-term policy advice for the global challenges of the coming century. The use of instability indicators drawn from the natural sciences may well provide critical insights into that process.

## Methods

### Data

Here we use three primary datasets. The Maddison Project historical real GDP per capita data in fixed 2011 US$^44^. The World Bank real GDP per capita data in 2018 US$^45^. The OECD real GDP data in 2011 US$^46^. The Maddison project and World Bank data are annual. The OECD data is quarterly.

### De-trending

**Figure 8:**
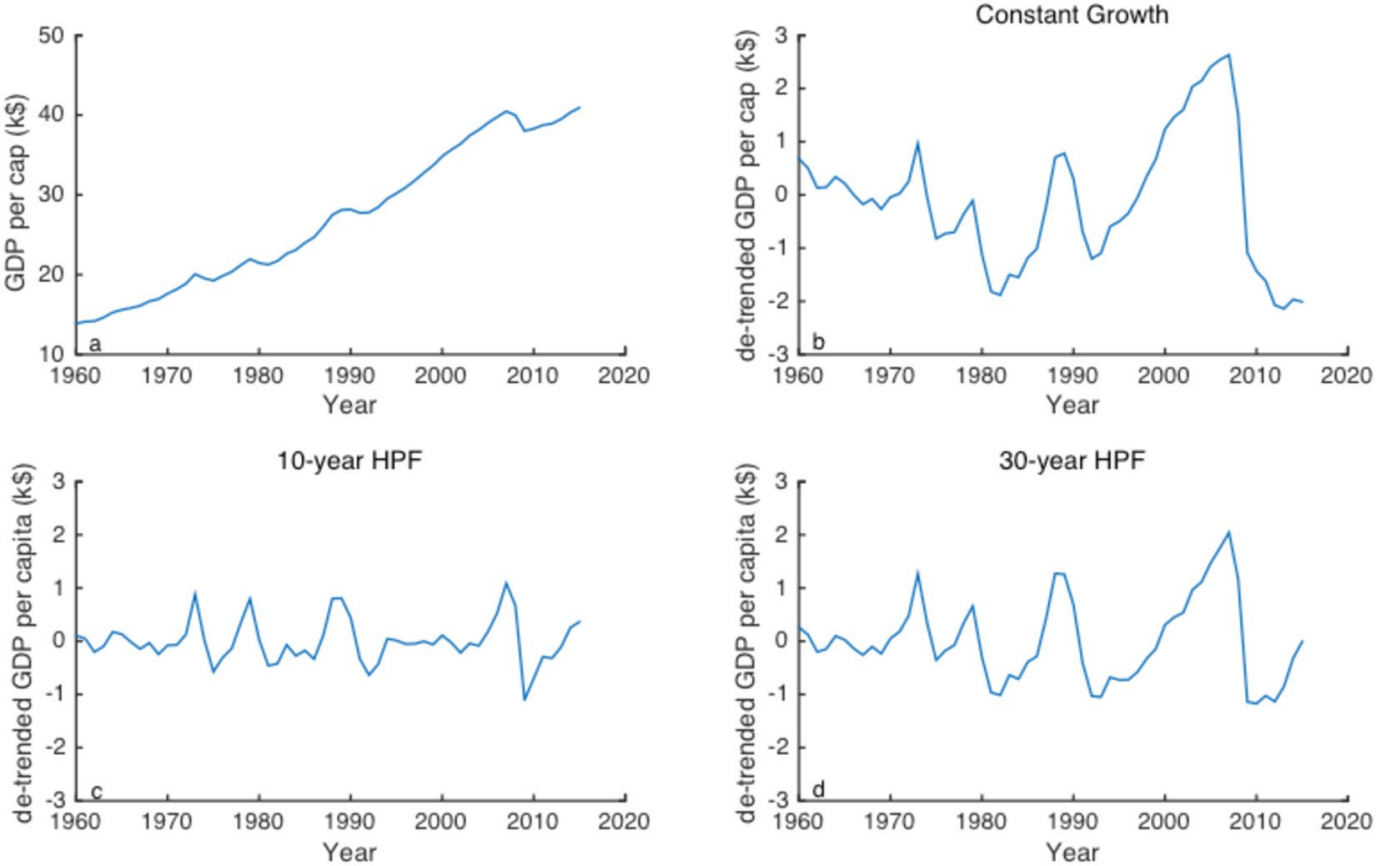
(**a**) UK GDP per capita 1955–2015, taken from the World Bank online data^65^; (**b)** Constant growth rate De-trended UK GDP per capita 1960–2015; (**c,d)** “10-year and 30-year, averaged growth” rate, HPF filter, De-trended UK GDP per capita, respectively.

Before analysis for CSD, time series must be de-trended to isolate variability about the growth path. Data are de-trended by subtracting a growth model (e.g. linear, exponential). The choice of growth model can have a large impact on results as shown in Fig. 8. Figure 8a shows the per capita GDP series for the UK. Figure 8b shows the same series de-trended by subtracting a linear growth rate. Figure 8c shows the same series de-trended using a conventional Hodrick-Prescott filter (HPF) with an averaging window of ~7–11 years (e.g. Lee 1955^64^). Figure 8d shows the same series de-trended using a HPF with a longer averaging window of ~30 years.

### Significance testing

**Figure 9:**
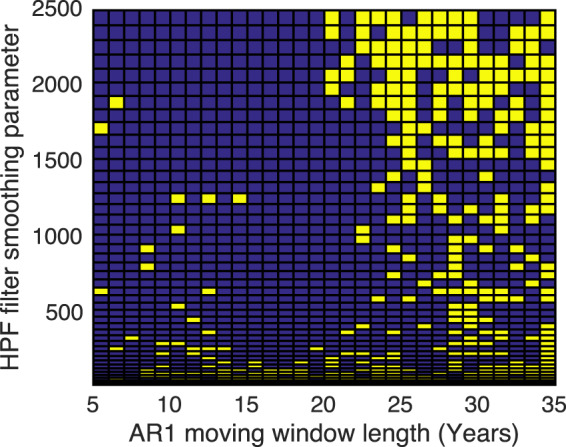
Parameter sweep matrix for the UK GDP per capita, AR1 calculation, using the Monte-Carlo significance test. Yellow regions show a combination of parameters that give insignificant AR1. Blue: significant.

The significance of trends in AR1 and variance (e.g. Figure 9) are tested using both Kendall Rank and Monte-Carlo tests, for a range of (de-trending, HPF filter) smoothing parameter values and ‘moving window’ sizes. A trend is determined to be significant if it satisfies p < 0.05 for the majority of calculation parameter values. Figure 9 shows the p matrix for the UK’s GDP-AR1 1960–2015 with a large range of parameter values.

## Supplementary information

Supplementary Information.

## Data Availability

The data used in this work is freely available. The Maddison Project dataset is available from: https://www.rug.nl/ggdc/historicaldevelopment/maddison/releases/maddison-project-database-2018. The World Bank datatset is available from: http://databank-.worldbank.org/. The OECD dataset is available from: https://data.oecd.org/.
