# Supplementary material for: Using critical slowing down indicators to understand economic growth rate variability and secular stagnation
Source: Sci Rep. 2020 Jun 26;10:10481. doi: 10.1038/s41598-020-66996-6 (PMC7320017; doi:10.1038/s41598-020-66996-6)
Supplement: Supplementary file 1 — Supplementary Information. [file 41598_2020_66996_MOESM1_ESM.pdf]

Using critical slowing down indicators to understand economic growth rate variability and secular stagnation

Supplementary material

Craig D. Rye[1*] and Tim Jackson[2]

1. Climate Science Awareness and Solutions, Columbia University (CSAS), New York, USA

2. Centre for the Understanding of Sustainable Prosperity (CUSP), University of Surrey, UK

Additional description of CSD

Diks Hommes and Wang (2015) use a simple, common example to introduce CSD behaviour. Consider a stochastically perturbed ball, rolling around a gravity. In this example, the gravity well can be thought of as the system's 'attractor space'. A system's attractor space describes both the range of behaviour that a system can express and the most likely behaviour that the system will express. The shape of the gravity well (attractor space) is dynamic, it changes shape as the restoring forces of the system change. A deep well represents a strong attractor with strong restoring forces and a shallow well represents a weak attractor with weak restoring forces. If the ball is randomly perturbed while in a deep well, it is rapidly restored to its equilibrium position (i.e. the bottom of the well). As the system becomes unstable, its local attracter space (the gravity well) becomes shallower then the ball oscillates further and more slowly around its equilibrium. If the well becomes increasingly shallow, then the ball becomes increasingly susceptible to falling into an adjacent well. As the system approaches its tipping point it undergoes CSD, and it spends an increasing amount of time in the new steady state. This type of transition is referred to as a saddle-node fold.

The concept of 'tipping points' is important to CSD research. In CSD research, a 'tipping point' occurs when a given perturbation is sufficient to move the system from one equilibrium (attractor well) to another. Tipping points are most likely to occur when the restoring forces of the local equilibrium are relatively weak.

A well cited example of a tipping point in the earth's climate system could be the rapid slowing of the Atlantic Meridional Overturning Circulation[43]. An example of a tipping point in macroeconomics could be associated with the response of an economy following a technological shock, or significant recession[20].

Additional results for the World Bank and OECD data

The World Bank and OECD data have a shorter time period (1960 to present) and are unable to provide historical insight beyond the 1960s. However, the World Bank dataset provides a broader range of economies compared to the Maddison Project dataset and the OECD dataset provides a higher temporal resolution. The following section provides some additional discussion of these complimentary datasets. The following section provides a list of the countries that express a positive trend in AR1 in the World Bank dataset, followed by example time series of AR1 for 9 economies in the world bank dataset and example time series of AR1 for 9 economies in the OECD dataset.

A list of the countries that express a significant positive trend in AR1 autocorrelation, in the recent, yearly per capita GDP data[46] (1960-2016): Algeria, Argentina, Australia, Bahamas, Bangladesh, Belgium, Benin, Bermuda, Burkina Faso, Burundi, Cameroon, Canada, Chad, Chile, China, Congo Dem. Rep., Cote d'Ivoire, Cuba, Denmark, Finland, France, Ghana, Greece, Guinea-Bissau, Guyana, Iceland, India, Indonesia, Italy, Kenya, Lesotho, Liberia, Mauritania, Nepal, Nicaragua,

Norway, Pakistan, Panama, Papua New Guinea, Peru, Puerto Rico, Senegal, Seychelles, South Africa, St. Vincent and the Grenadines, Seychelles, Swaziland, Sweden, Togo, Tunisia, United Kingdom, United States, Uruguay, Zambia, Zimbabwe.

It is noteworthy that this group contains a 7 of the 10 largest economies (by nominal GDP). It represents approximately 60% of world (nominal) GDP. Further, it contains many small economies synonymous with tax evasion, such as the Bahamas, St. Vincent and the Grenadines, Seychelles and Panama.

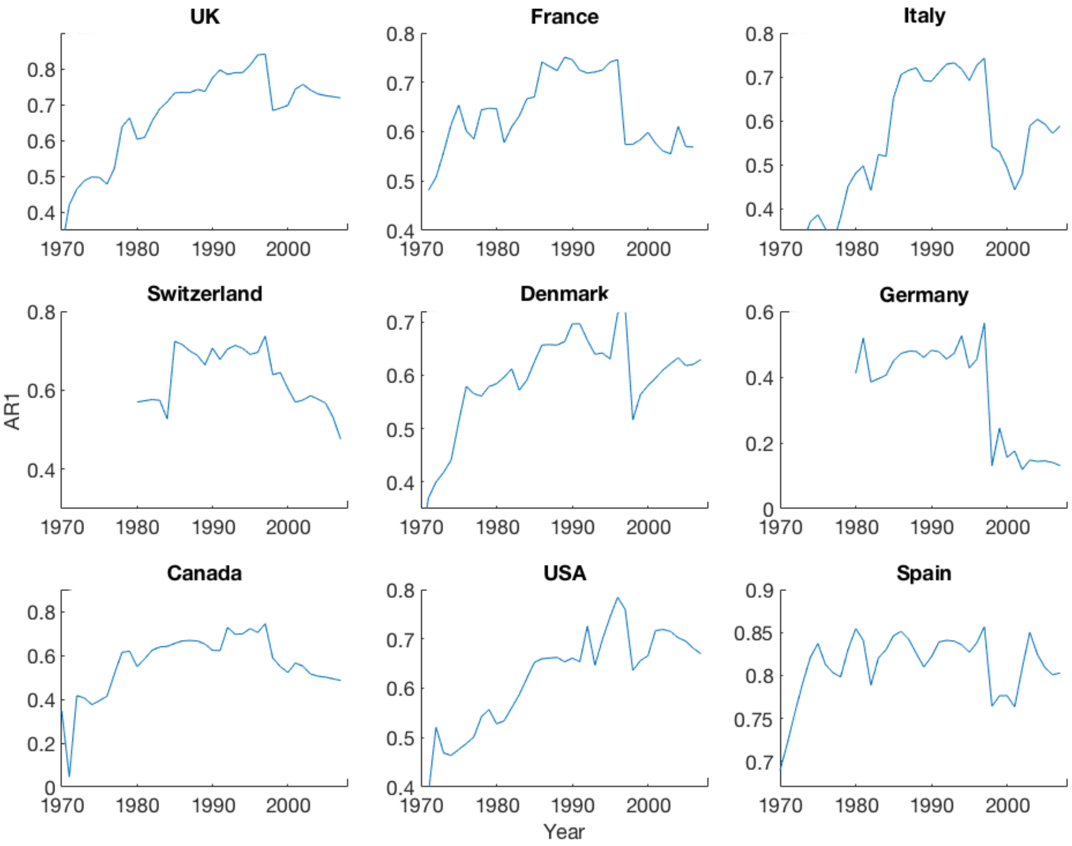

Figure S1 | AR1 autocorrelation derived from annual de-trended real GDP per capita data between 1900-2060 (2010 US$ prices), taken from the World Bank[47].

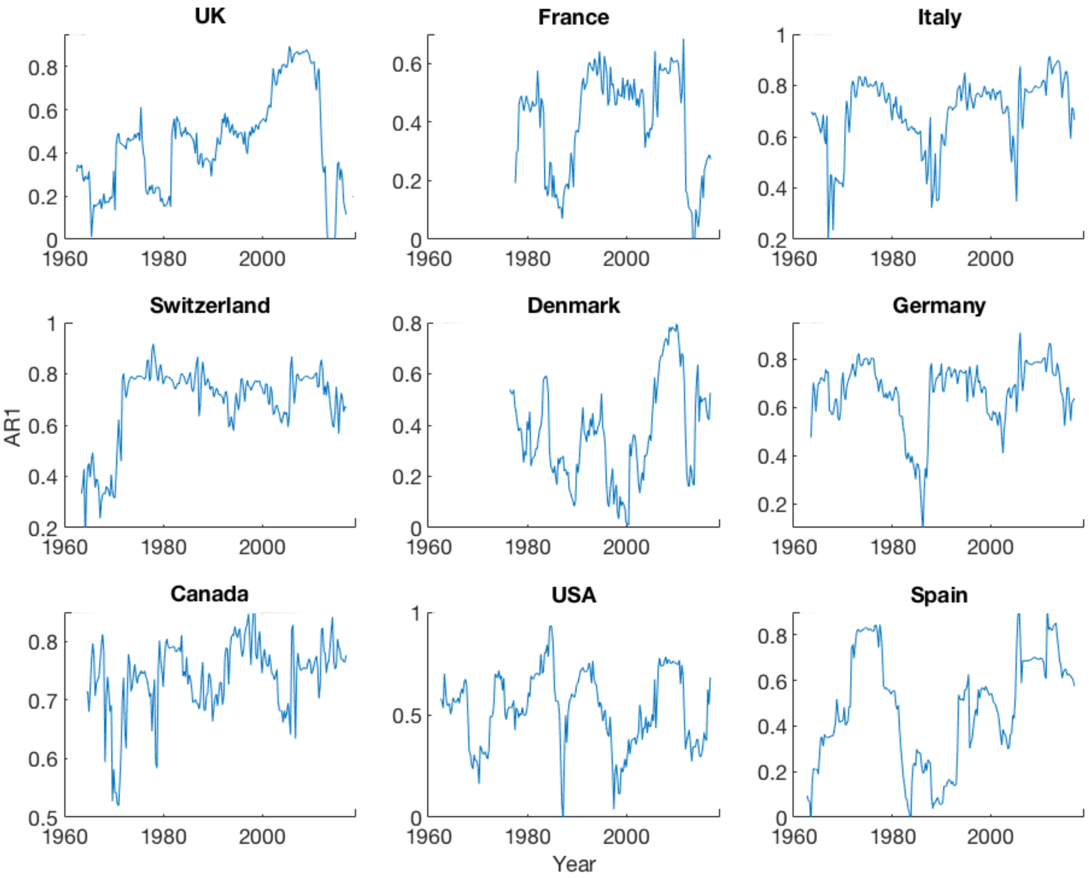

Figure S2 | AR1 autocorrelation derived from de-trended quarterly real GDP data between 1900-2060 (2018 US$ prices), taken from the OECD[48].
